# Structural insights into thrombolytic activity of destabilase from medicinal leech

**DOI:** 10.1038/s41598-023-32459-x

**Published:** 2023-04-24

**Authors:** Egor Marin, Daniil A. Kornilov, Sergey S. Bukhdruker, Vladimir A. Aleksenko, Valentin A. Manuvera, Egor V. Zinovev, Kirill V. Kovalev, Mikhail B. Shevtsov, Anna A. Talyzina, Pavel A. Bobrovsky, Pavel K. Kuzmichev, Alexey V. Mishin, Ivan Y. Gushchin, Vassili N. Lazarev, Valentin I. Borshchevskiy

**Affiliations:** 1 Moscow Institute of Physics and Technology, Dolgoprudny, Russia; 2 Lopukhin Federal Research and Clinical Center of Physical-Chemical Medicine of Federal Medical Biological Agency, Moscow, Russia; 3 EMBL Outstation Hamburg, c/o DESY, Hamburg, Germany; 4 Joint Institute for Nuclear Research, Dubna, Russia; 5 Present Address: Department of Molecular Biosciences, Northwestern University, Evanston, IL USA; 6 Present Address: Groningen Biomolecular Sciences and Biotechnology Institute, University of Groningen, Groningen, The Netherlands

**Keywords:** X-ray crystallography, Molecular modelling

## Abstract

Destabilase from the medical leech *Hirudo medicinalis* belongs to the family of i-type lysozymes. It has two different enzymatic activities: microbial cell walls destruction (muramidase activity), and dissolution of the stabilized fibrin (isopeptidase activity). Both activities are known to be inhibited by sodium chloride at near physiological concentrations, but the structural basis remains unknown. Here we present two crystal structures of destabilase, including a 1.1 Å-resolution structure in complex with sodium ion. Our structures reveal the location of sodium ion between Glu34/Asp46 residues, which were previously recognized as a glycosidase active site. While sodium coordination with these amino acids may explain inhibition of the muramidase activity, its influence on previously suggested Ser49/Lys58 isopeptidase activity dyad is unclear. We revise the Ser49/Lys58 hypothesis and compare sequences of i-type lysozymes with confirmed destabilase activity. We suggest that the general base for the isopeptidase activity is His112 rather than Lys58. pKa calculations of these amino acids, assessed through the 1 μs molecular dynamics simulation, confirm the hypothesis. Our findings highlight the ambiguity of destabilase catalytic residues identification and build foundations for further research of structure–activity relationship of isopeptidase activity as well as structure-based protein design for potential anticoagulant drug development.

## Introduction

Hirudotherapy, or medicinal leech therapy, is an old technique used for medicinal purposes for millennia and continues to be used today in modern hospitals for various diseases such as inflammatory and cardiovascular diseases, thrombosis, and after different surgeries^1^. For this reason, the European medicinal leech, *Hirudo medicinalis*, is currently subjected to intense genome mining^2,3^ as a potential source of bioactive molecules that can be developed as drug leads.

The main therapeutic effects of hirudotherapy come from extremely potent anticoagulation factors secreted by the leech into the wound during feeding. While other known factors prevent thrombi formation, destabilase is the only one has a fibrinolytic effect^4^.

Destabilase from the medicinal leech (*Hirudo medicinalis*) is the first described and well-characterized member of polyfunctional lysozymes from invertebrates (i-type lysozymes)^5^. Originally, the enzyme was extracted from salivary gland secretion of medicinal leech in a composition of a liposomal complex^5^. Destabilase combines muramidase (lysozyme) (EC 3.2.1.17), endo-ε-(γ-Glu)-Lys-isopeptidase (EC 3.5.1.44) and non-enzymatic antibacterial activities^6,7^. Destabilase is also known to have robust antimicrobial properties: the enzyme with heat-inactivated muramidase activity possesses toxic effect against fungi (*Botrytis cinerea*) and yeast (*Candida guilliermondii* and *Schizosaccharomyces pombe*)^8^, as well as gram-positive (*Micrococcus luteus*) and gram-negative (*Escherichia coli*) bacteria^6^. Moreover, some synthetic amphipathic fragments of destabilase were shown to have antimicrobial activity^6^.

Isopeptidase activity of destabilase manifests itself in the form of a specific hydrolysis of ε-(γ-Glu)-Lys isopeptide bonds (bonds between side-chains of Glu and Lys) in the absence of peptidase function for α-(α-Glu)-Lys peptide bonds^9^. This enzymatic activity of destabilase is usually detected by cleavage of the ε-(γ-Glu)-Lys dipeptide or its synthetic analog L-γ-glutamine-p-nitroanilide (L-γ-Glu-pNA)^10,11^. Such ε-(γ-Glu)-Lys isopeptide bonds are formed by specific transglutaminases and play an important role in many vital processes^12^, such as blood coagulation mediated by Factor(XIIIa). In this process the covalently stabilized, insoluble fibrin clot is generated by cross-linking of γ- and α-chains of separate fibrin molecules^13^. Due to its isopeptidase activity destabilase dissolutes stabilized fibrin and cleaves the bond formed between side chains of lysine and glutamine residues in the D-dimer (the final product of fibrinolysis)^9^. Predictably, destabilase greatly increases the thrombolysis of both venous and arterial thrombi in rat model^4^ and destroys old, preliminarily formed thrombi in in vitro and in vivo experiments^11,14^. This ability makes destabilase a promising thrombolytic drug candidate with reduced side effects and increased effectivity^4^. Despite evident importance, the structure of destabilase remains unrevealed until now.

Destabilase remained the only known i-type lysozyme with isopeptidase activity until a number of similar lysozymes were found in other invertebrates (Table 1). Among them are enzymes isolated from the annelid worm (*Eisenia andrei*)^15^, the sea cucumber (*Apostichopus japonicus*)^16^, the purple sea urchin (*Strongylocentrotus purpuratus*)^17^, the sea snail (*Haliotis discus discus*)^18^ and the saltwater clam (*Tapes japonica*)^19^.

**Table 1:** Occurrence of muramidase and isopeptidase activities among destabilase homologs.

Host	UniProt ID	Muramidase activity	Isopeptidase activity	References
*Haliotis discus discus*	U5KC58	+	+	^18^
*Tapes japonica*	Q8IU26	+	+	^19^
*Eisenia andrei*	Q0ZME1	+	+	^15^
*Apostichopus japonicus*	A0MT08	+	+	^16^
*Strongylocentrotus purpuratus*	A0A7M7TGP5	+	+	^17^
*Procambarus clarkii*	A0A346QR85 (Pclysi1)	−	−	^39^
	N/A (Pclysi2)	−	+	
*Scylla paramamosain*	A0A1W5RN49	−	+	^61^
*Meretrix meretrix*	F6JX82	+	N/A	^62^
*Sinonovacula constricta*	A0A513U825	+	N/A	^63^
*Crassostrea virginica*	P83673 (cv-lysozyme 1)	+	−	^64^
	Q1XG90 (cv-lysozyme 2)	+	−	
*Harmonia axyridis*	A0A0S1TQ24	−	−	^65^
*Cristaria plicata*	I6ZIX7	+	N/A	^66^
*Asterias rubens*	Q6TP50	+	N/A	^67^
*Penaeus monodon*	D2J087	−	N/A	^68^
*Meretrix lusoria*	P86383	+	N/A	^69^
*Hirudo medicinalis*	Q25091	+	+	^7^

Isopeptidase activity was confirmed via cleavage of L-γ-Glu-pNA and muramidase activity—via the destruction of *Micrococcus lysodeikticus* cellular walls.

Isopeptidase activity of destabilase as well as of *Tapes japonica* lysozyme (TjL) is inhibited by the irreversible inhibitors of serine proteases, PMSF (phenylmethylsulfonyl fluoride) or AEBSF (4-(2-Aminoethyl)benzenesulfonyl fluoride), suggesting that Ser is involved in isopeptidase activity^19,20^ and thus its active site is similar to those of serine proteases. Previously, basing on weak sequence homology with serine proteases, Ser49 or Ser51 of destabilase were proposed to be involved in isopeptidase activity^20^. Other engaged amino acids were assumed to be Lys58, Lys59, Ser82 and His112, with the last two being conserved in homologous TjL with confirmed isopeptidase activity^19^.

Further 3D homology modeling followed by site-directed mutagenesis revealed that functional centers of isopeptidase and muramidase activities are located close to each other with Ser49/Lys58 responsible for isopeptidase and Glu34/Asp46 for muramidase activity^21^. However, the molecular mechanism for these activities remains unknown.

In the present study, we further explore structural bases of destabilase activities. We report the first high-resolution crystallographic structures of destabilase from *Hirudo medicinalis*, obtained at low sodium concentration at pH 8.0 and high sodium concentration at pH 5.0 with a resolution of 1.4 Å and 1.1 Å, correspondingly. We further discuss the role of the sodium ion as a muramidase and isopeptidase activity inhibitor. Finally, based on phylogenetic analysis supported by molecular dynamics calculations we propose that His112 rather than Lys58 may serve as a general base of the isopeptidase catalytic center.

## Results and discussion

### Phylogenetic analysis

**Figure 1:**
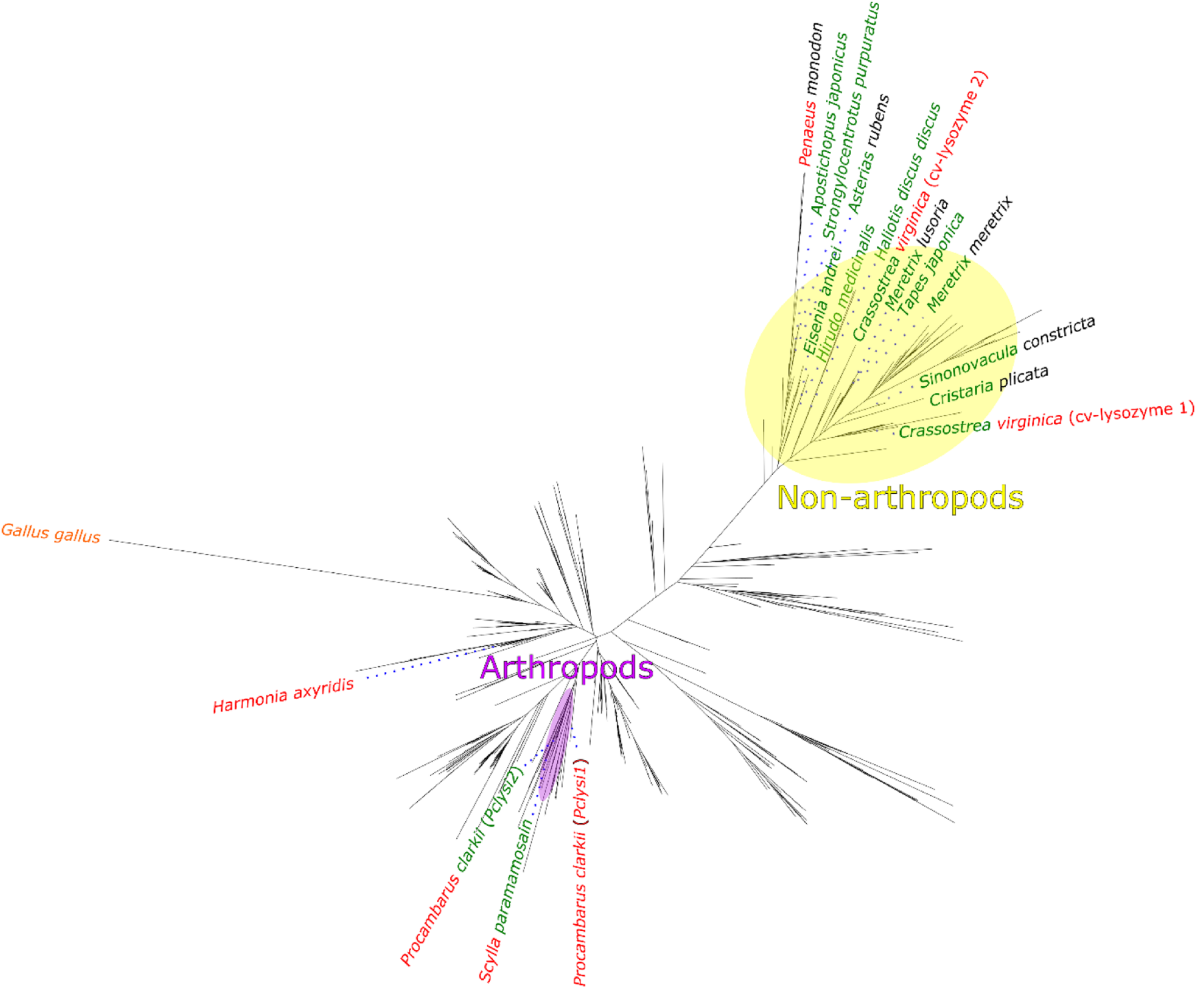
Phylogenetic analysis of Pfam “destabilase” protein family, annotated with information about muramidase and isopeptidase activities and source organisms. An outgroup protein (c-type lysozyme from hen egg *Gallus gallus*, colored with orange*)* is added as a distant relative of i-type lysozymes, indicating the root of the phylogenetic tree. A color of the first half of the organism taxonomy name corresponds to the muramidase activity and the second half—to the isopeptidase activity. Green and red colors mean presence and absence of the corresponded activity respectively, black means no information about the corresponding activity. Violet shows arthropods branch of the tree, yellow—the non-arthropods branch.

To get evolutionary insight into relationships between the various i-type lysozymes, we have constructed a phylogenetic tree using the Pfam database^22^ (Fig. 1) and plotted on the tree available data about muramidase and isopeptidase activities (Table 1). Lysozymes with isopeptidase activity belong to two distinct branches of the phylogenetic tree: arthropods’ and non-arthropods’ branches.

**Figure 2:**
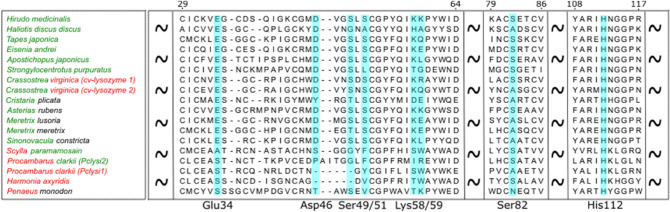
Multiple sequence alignment of destabilase homologs with confirmed muramidase and/or isopeptidase activities from Table 1. Color of the first half of the organism taxonomy name corresponds to the muramidase activity and the second half—to the isopeptidase activity. Green and red colors mean presence and absence of the confirmed corresponding activity, black means no information is available related to corresponding activity. Amino acids discussed in the manuscript are marked with light blue.

Lysozymes with isopeptidase activity are expected to have a conserved Ser in the active site. The only conserved serine residue in proteins from both branches is Ser82 (here and below we refer to immature destabilase amino acid residues numbering, UniProt ID: Q25091) (Fig. 2). This serine residue was historically the first candidate suspected of belonging to the isopeptidase active site^20^. However, as we show below, Ser82 is buried deep in the protein core and has no access to any protein cavities, which contradicts its engagement in the isopeptidase activity.

The non-arthropods’ group is the larger among the two and contains 6 lysozymes with confirmed isopeptidase activity including destabilase. They have conserved Ser49 and/or Ser51 with the sporadic exception of *Haliotic discus discus* (Fig. 2). These serine residues were previously suggested as the main candidates for catalytic serines of isopeptidase activity^21^. Notably, many other lysozymes from the non-arthropod branch with untested isopeptidase activity have conserved Ser49 and/or Ser51. Those are primary candidates to be examined as serine isopeptidases in future studies.

The arthropods’ group is smaller and contains lysozymes with confirmed isopeptidase activity from *Procambarus clarkii (Pclysi2)* and *Scylla paramamosain*. These proteins have neither conserved Ser49 nor Ser51. Therefore, non-arthropods and arthropods probably obtained their isopeptidase activity independently during the evolution. In addition, the isopeptidase activity of arthropods may be of non serine protease origin.

### Overall structure of *H. medicinalis* destabilase

To get structural insights into destabilase function we solved its structure in two different crystal forms: P1—obtained at pH 5.0 and high sodium salt concentrations (2.9 M sodium malonate) and P2_1_—obtained at pH 8.0 and low sodium concentration (0.3 M sodium chloride). Further on, we refer to these structures as high and low salt structures, correspondingly.

**Figure 3:**
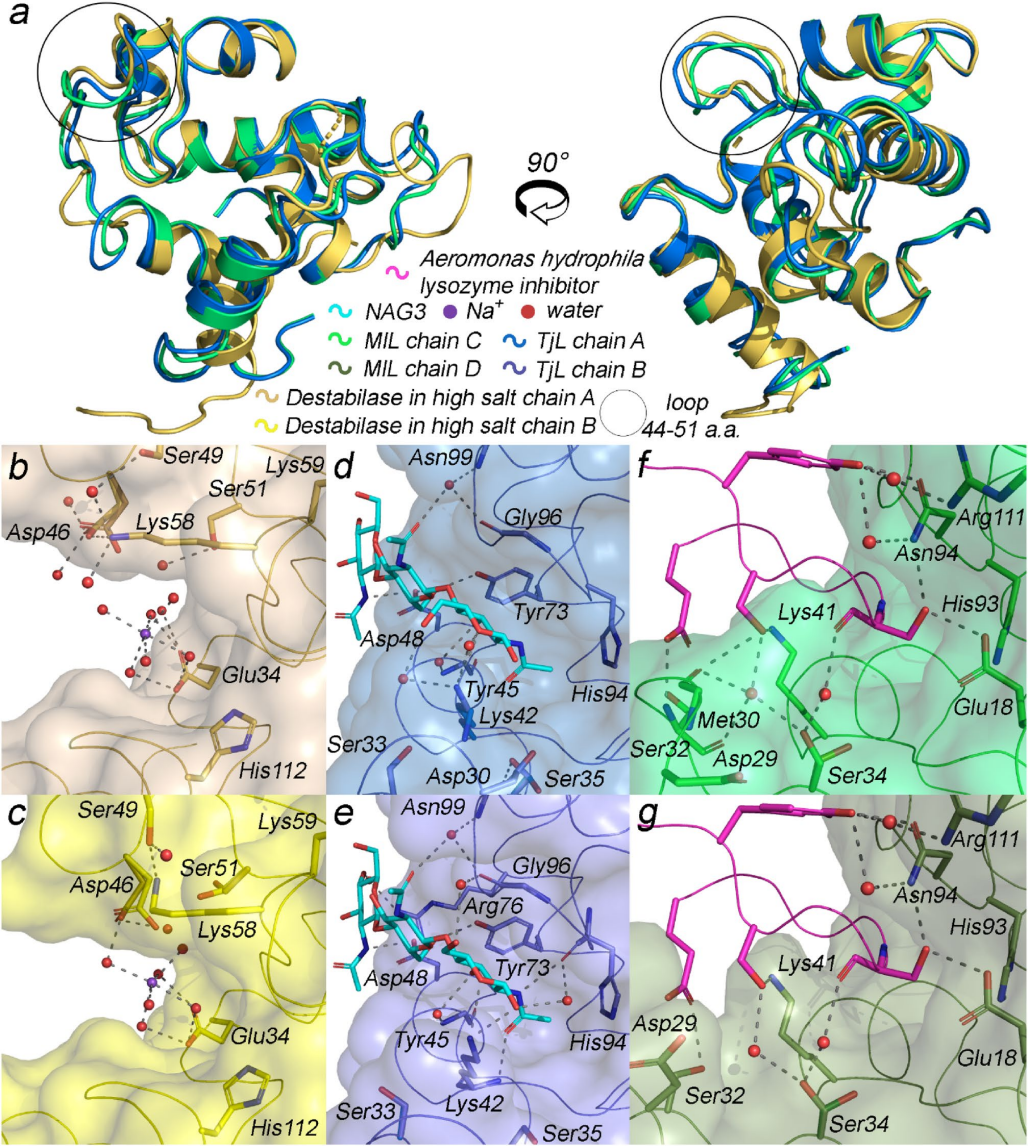
High salt destabilase structure and of its homologs. (**a**) Overall structure superposition of high salt destabilase chain A, TjL chain A (PDB ID 2DQA^23^) and MlL chain C (PDB ID 4PJ2^24^); lateral view on the inhibited enzymatic centers of high salt destabilase ((**b,c**) for chains A, B, correspondingly), TjL ((**d,e**) for chains A, B, correspondingly) and MlL ((**f,g**) for chains C, D, correspondingly). Residues potentially responsible for muramidase and isopeptidase activities or bonded to the inhibitor through the water molecules are shown as sticks. Inhibitors of muramidase activity are depicted as spheres, sticks or ribbon diagrams, respectively: (**b,c**) water coordinated Na^+^ ion; (**d,e**) NAG3; (**f,g**) *Aeromonas hydrophila* lysozyme inhibitor. Water and inhibitory molecules as well as hydrogen bonds are shown for alternative conformation A only in MlL and TjL structures and for alternative conformations with presence of sodium in destabilase structures. The variability of an inhibitor-bound TjL and MlL loop 44–51 confirms the importance of the loop flexibility for enzymatic activities of the proteins.

Both destabilase structures share overall structure with the published homologous TjL (PDB ID: 2DQA^23^) and *Meretrix lusoria* lysozyme (MlL, PDB ID: 4PJ2^24^): C_α_–C_α_ RMSD between all four structures is not exceeding 2 Å (Fig. 3).

The DALI program^25^ revealed that in addition to mentioned lysozymes a number of lytic transglycosylases (LTs) (PDB IDs: 7LAM^26^, 4C5F^27^, 4OWD, 6FPN^28^, 1QTE^29^) share high structural similarity with destabilase. Similar to lysozymes, LTs have substrate specificity for the glycosidic linkage of peptidoglycans. While lysozymes act as hydrolytic catalysts, LTs facilitate non-hydrolytic scission required for cell wall transformations in host bacteria^30^.

One of the unique features of destabilase is the high number of cysteine residues. While in average cysteines accounts for 1.38% of all amino acid residues in proteins^31^, destabilase has 14 cysteines per 115 total residues. All of them form disulfide bonds and are arranged in a close agreement with other i-type lysosomes TjL and MlL.

**Figure 4:**
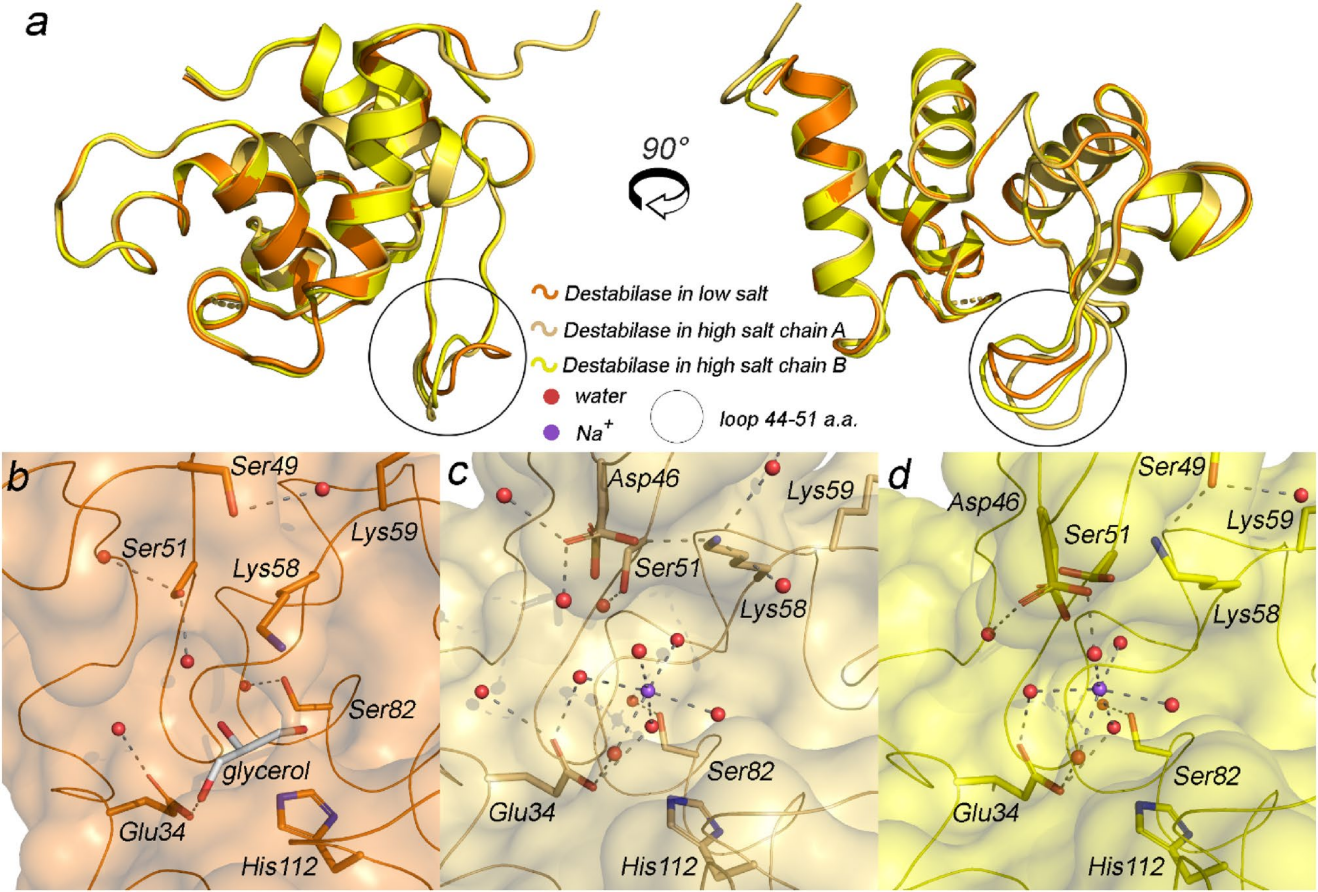
High and low salt destabilase structures. (**a**) Overall structure superposition of all destabilase structures; (**b**) front view of the catalytic cleft in the low salt destabilase structure; (**c,d**) front view of the catalytic cleft in the high salt destabilase structure in chains A and B, correspondingly; residues, potentially responsible for either muramidase or isopeptidase activity, are shown as sticks; Na^+^ ion and water molecules are shown as spheres. Water molecules and hydrogen bonds are shown only for alternative conformations with presence of sodium. Water coordinated Na^+^ ion occupies the cleft between catalytic dyad Glu34/Asp46 and fixates the flexible loop 44–51 in the closed conformation preventing a substrate from approaching the active site.

The asymmetric unit contains two protein molecules in essentially the same conformation (C_α_-C_α_ RMSD equals 0.63 Å) in the case of high salt conditions, while one protein molecule is present per asymmetric unit in low salt structure (Fig. 4).

### Structural basis for inhibition of muramidase activity by sodium ions

The C_α_–C_α_ RMSD between the low salt and both chains of high salt structures is less than 0.8 Å. The main difference between low and high salt structures as well as between the two chains of high salt structure is tied to a flexible loop (Gly44-Ser51), containing enzymatically important Asp46 (Fig. 4a). Together with Glu34, Asp46 forms a catalytic dyad for muramidase activity in i-type lysozymes^23^. In the low salt structure, the Gly44-Ser51 loop is bent outward with Asp46 exposed to the bulk solution (Fig. 4b). Val47 backbone forms a H-bond with an adjacent protein molecule, and therefore crystal packing contributes to stabilization of “open” conformation of the Gly44-Ser51 loop. In the high salt structure, both protein chains possess a more compact Gly44-Ser51 loop adherent to the protein core (Fig. 4c,d). Asp46 has two alternative conformations in both chains and in all cases is oriented towards Glu34.

Both isopeptidase and muramidase activities are inhibited by submolar sodium chloride concentrations^32^. In our high salt structure, both A and B chains contain one sodium ion with 50% occupancy situated in the cleft between Glu34 and Asp46 and stabilized in octahedral coordination with water molecules by Glu34 and Asp46 side chains (Fig. 3b,c). Our low salt structure lacks the sodium ion but contains cryoprotectant glycerol molecule, H-bonded with Glu34 but not Asp46 (Fig. 4b–d).

Notably, the same binding cavity is occupied by the muramidase product inhibitor (Tri(*N*-acetyl-D-glucosamine) (NAG3)) and proteinaceous lysozyme inhibitor in TjL (PDB ID: 2DQA^23^) and MlL (PDB ID: 4PJ2^24^) structures, respectively (Fig. 3d–g). Both structures have two lysozyme chains, which differ in the conformation of homologous Gly44-Ser51 loop. The inhibitor-bound MlL has more open loop conformations, while inhibitor-bound TjL has more closed ones. Such variability points out to the importance of the loop conformational flexibility for capturing the substrate in i-type lysozymes. The inhibitor (whether it is sodium ion in destabilase or NAG3 in TjL) fixes the loop in the closed conformation; this explains the inhibition mechanism.

Remarkably, in addition to its high sodium content, the crystallization sample of the high salt destabilase structure also included a muramidase product inhibitor NAG3. However, electron density maps did not reveal the presence of NAG3 molecule, suggesting that the inhibitory effect of sodium ions was at play. Interestingly, both sodium ions and NAG3 were present in the crystallization conditions of TjL (PDB ID: 2DQA^23^) and MlL lysozyme (PDB ID: 3AB6^33^). In contrast to our study, these structures contained NAG3 molecules but no sodium ions. This discrepancy may be due to lower sodium concentrations in these structures, which were not sufficient to produce an inhibitory effect under the crystal conditions.

### Determination of catalytic residues of isopeptidase activity in destabilase

Serine proteases are the best-known class of proteases^34,35^, which classically contain Ser/His/Asp catalytic triad. Here serine is the nucleophile, histidine is the general base, and aspartate is the acid, helping to orient the histidine residue and neutralize the charge that develops on the histidine during the transition states. However, there are a number of atypical serine proteases which use alternative general base (such as Glu) and acid (Glu or His) or have catalytic dyad instead of triad with Lys or His as a general base^34,36^. In some cases, an individual Ser may serve as the only catalytic residue^34^.

As we have already mentioned in the phylogenetic analysis section, there are three Ser residues, conserved in the non-arthropod branch: Ser49, Ser51 and Ser82. The latter one is buried in the protein, while Ser49/51 are located in the protein cleft and, therefore, are the main candidates for the nucleophile of isopeptidase activity. Given that the sodium ion appears near Ser51 in our high salt structure, we propose this serine to be the nucleophile in destabilase.

General base residue accepts and releases a proton during protease catalytic cycle. Therefore, it is expected to have pK_a_ close to the pH optimum of the enzyme activity, which is between 5 and 7 for destabilase^34^. As we mentioned above, His, Glu and Lys may serve as a general base. There are 4 amino acids of these types in the vicinity of Ser49/51: Glu34, Lys58, Lys59, His112. Additionally, Asp46 may also be a candidate due to its chemical similarity to Glu.

**Figure 5:**
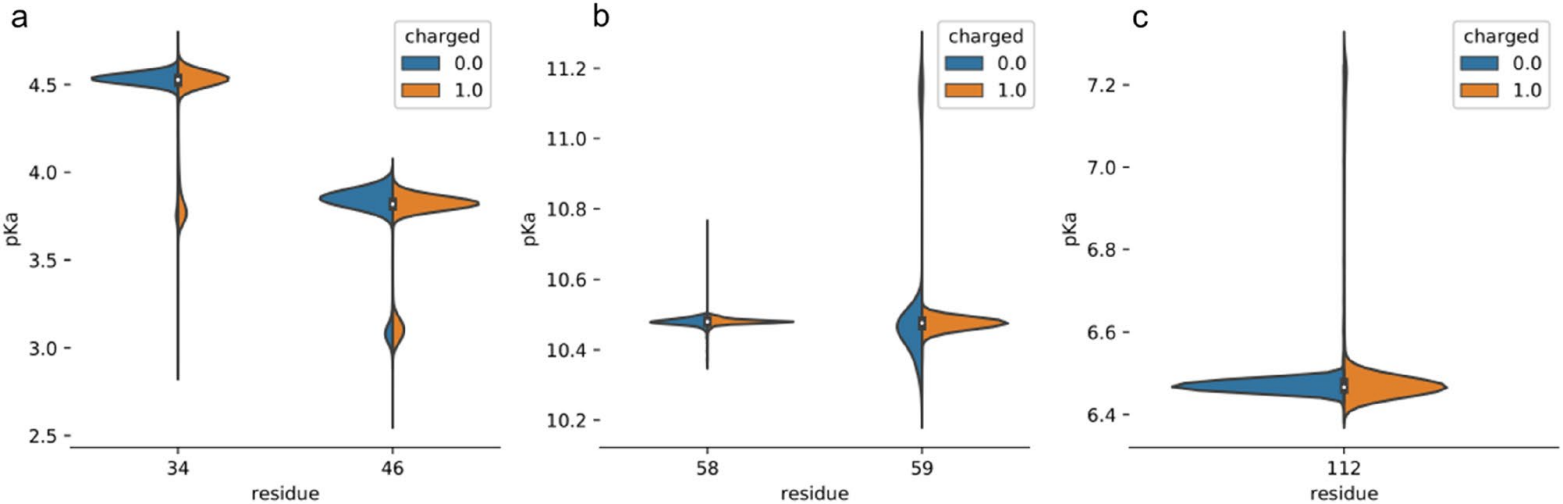
Structure-based pK_a_ values, calculated over the 1 μs molecular dynamics simulations of the destabilase (high salt structure). (**a**) Glu34 and Asp46 residues, (**b**) Lys58 and Lys59 residues, (**c**) His112 residue. Blue and orange violin plots represent uncharged and charged His112 trajectories, respectively. Plot width is proportional to the total share of structures with the respective pKa value.

To determine the general base residue among the 5 possible candidates, we performed µs-long unbiased molecular dynamics simulations and estimated pK_a_ of these residues at each frame of the simulation (Fig. 5). Both Lys58 and Lys59 have pK_a_’s above 10.0 in all accessible conformations. These residues are solvent-exposed and thus their pK_a_ are close to the pK_a_ of a free lysine in solution. pK_a_’s of carboxylic residues Asp46 and Glu34 are both below 5. In contrast, our molecular dynamics simulations predict the pK_a_ of His112 to be around 6.4, which is within the activity range of the enzyme.

Thus, we showed that Ser51 is the most probable nucleophile, while His112 is the general base. The only possible candidate for the acid is Glu34 situated in the immediate vicinity of the His112. Thus, we presume the isopeptidase active site of destabilase to have an architecture similar to the Ser-His-Glu triad of serine proteases.

Previously, *Zavalova *et al. suggested that Ser49-Lys58 dyad, but not Ser51-His112-Glu34 triad, realizes isopeptidase activity^21^. Their conclusions are based on the 3D homology model of destabilase and mutagenesis analysis. This is not confirmed by our results. Destabilase is expressed in soluble form in human Expi293F cell line in our study, while it was refolded from *E.coli* inclusion bodies by *Zavalova *et al.^21^. The direct comparison showed (see Fig. 6 in Ref.^37^) that the latter approach results in a protein with extremely low isopeptidase activity and with circular dichroism (CD) spectra of denatured protein, while the protein from Expi293F shows high activity and reasonable CD spectra. Therefore, we suggest that the contradiction originates from low functional expression in *E.coli* and mutagenesis experiments should be reproduced with the protein obtained from Expi293F cell line.

The functional triads are highly stabilized by mutual hydrogen bonds in the large majority of serine protease structures with very strong low-barrier hydrogen bonds observed in several cases^38^. Surprisingly, all three amino acid residues in the isopeptidase site of destabilase do not form hydrogen bonds to each other in both high and low salt structures (Fig. 4). We expect it is because both our structures were substrate-free. Given that Ser51 residue is located in the flexible loop, in a substrate-bound state it can approach His112/Glu34, His112 side chain can flip and all necessary hydrogen bonds can be formed.

## Conclusions

In the present work we performed phylogenetic analysis of i-type lysozymes from the Pfam database and showed that isopeptidase activity emerged independently in arthropod and non-arthropod branches. Since inhibitors of serine proteases can also inhibit isopeptidase activity of the lysozymes from the non-arthropod branch^19,20^, we propose three conserved Ser residues as candidates for nucleophile of the catalytic active site.

We obtained the first high-resolution X-ray structures of the destabilase in high and low salt conditions at 1.1 and 1.4 Å respectively. These structures provide structural basis for the inhibition of muramidase and isopeptidase activities: a sodium ion occupies the cleft between catalytic dyad Glu34/Asp46 and functionally flexible loop Gly44-Ser51 prevents the substrate from approaching the active site.

New structural information presented here also shows Ser51 to be the most probable nucleophile for isopeptidase activity. Further molecular dynamics simulation with the new structure demonstrates that His112 could serve as the general base instead of the previously proposed Lys58. Our results suggest that together with the adjacent Glu34 and His112, Ser51 could form a Ser-His-Glu active triad for the isopeptidase activity.

Thus, the present study provides structural basis and mechanistic insights into the isopeptidase activity of destabilase. Our findings lay a foundation for further research of the structure–activity relationship of isopeptidase activity as well as structure-based protein design for potential anticoagulant drug development.

## Materials and methods

### Phylogenetic analysis

Sequence set for analysis was collected from the family PF05497 of Pfam database^22^, release 33.1 at the moment of retrieval (December 2020). Alignment of 936 sequences’ fragments from PFAM was used as a base alignment. Bacterial and viral metagenomic sequences together with several other sequences were grouped in the same branch. This branch was excluded from the analysis since it had low support values and did not contain sequences meaningful for the analysis. Two additional sequences were added manually: P00698 (*Gallus gallus*) as an outgroup representative and Pclysi2 (*Procambarus clarkii*) as a sequence not present in the Pfam dataset but previously described in literature^39^.

MAFFT v7.475^40^ was used to align additional sequences to the Pfam alignment with—add option and L-INS-i algorithm. For phylogenetic tree estimation, TrimAl^41^ 1.2 was used to remove columns in alignment with gap content more than 90%. Phylogenetic trees were generated using RAxML^42^ version 8.2.12 using the PROTGAMMALG model executed on the Cyberinfrastructure for Phylogenetic Research (CIPRES) portal^43^. 20 alternative trees were used as starting points, with 12,345 as the random seed value. The tree was drawn in Figtree^44^ (version 1.4) with default parameters.

### Protein expression and purification

The preparation of recombinant destabilase was described in detail earlier^37^. Briefly, DNA fragments encoding destabilase isoform Ds2 (UniProt ID Q25091) were synthesized from oligonucleotides. The plasmid pcDNA3.4-Dest2 for the expression of the destabilase gene in the human cell line Expi293F was constructed using a pcDNA3.4-TOPO TA Cloning Kit (Invitrogen, USA). The transient human cell line Expi293F producing destabilase was generated using an Expi293F Expression System Kit (Life Technologies, USA). The cells were transfected by ExpiFectamine 293 transfection agent with pcDNA3.4-Dest2 plasmid followed by incubation for 120 h, the enzyme was secreted into the culture medium. The presence of C-terminal 6xHisTag allowed for the concentration and purification of the target protein using metal chelate chromatography on Ni Sepharose High Performance column (GE Healthcare). The yield of pure destabilase was 20 mg per 1 l of the human cell line culture. Additional purification step was performed using CM Sepharose Fast Flow media (GE Healthcare, USA). The solution of purified destabilase was dialyzed (SnakeSkin Dialysis Tubing 3.5 K MWCO, Thermo Fisher Scientific) against 5 mM Na_2_HPO_4_ at pH 5.0.

### Crystallization

The protein was concentrated by Amicon® Ultra 10 kDa up to 12 mg/ml prior to crystallization. The crystallization was set up in Corning® 3550 plates vapor diffusion plates using the NT8 nanovolume robot (Formulatrix, USA). In each well the drops contained 200 nl of concentrated protein solution and added reservoir solution in 3 different sample:precipitant ratios (2:3, 1:1 and 3:2). Best high salt crystals were obtained using 2.9 M sodium malonate pH 5.0 6.4 mM NAG3 (TCI Chemicals, CAS 38864-21-0), and best low salt crystals were obtained using 0.01 M Tris–HCl pH 8.0, 0.3 M NaCl, 27.5% w/v PEG 4000, 72 mM γ-Glu-ε-Lys dipeptide (Sigma, CAS 17105-15-6). All crystals (Supplementary Fig. 1) were grown at room temperature (20 °C) and reached their final size within 2 weeks (Supplementary Fig. 1).

### Crystal harvesting and crystallographic data collection

For the data collection, crystals were cryoprotected with 1:4 glycerol:well solution, harvested using MicroMount loops (MiTiGen) and flash frozen in the liquid nitrogen.

Data collection was performed at ID23-1 and ID-29 of the European Synchrotron Radiation Facility (ESRF, Block Allocation Group MX-2079: Russian BAG for Xtallography and BioSAXS), Grenoble, France, for the high and low salt structures, correspondingly.

### Crystallographic data processing and structure refinement

All the data were processed in the XDS software package^45^. The phase problem was solved by molecular replacement in phenix.phaser^46,47^, where the generated poly-ala model of TjL (PDB ID 2DQA^23^) was used as a starting model. Crystals at high salt conditions have a P1 space group with two molecules per asymmetric unit, while crystals at low salt conditions have a P2_1_ space group with one molecule per asymmetric unit. The models were subsequently rebuilt in phenix.autobuild^48^, phenix.refine^46^ and COOT^49^. TLS refinement was used in low salt destabilase structure. Final resolution cut-off was determined by paired refinement^50^. The quality of the resulting models was analyzed by phenix.molprobity^51^ and Quality Control Check web server^52^. Crystallographic data collection and refinement statistics are given in Supplementary Table 1. The figures were generated using PyMOL (The PyMOL Molecular Graphics System, Version 2.0 Schrödinger, LLC)^53^.

Three sodium ions were identified in the high salt destabilase structure (atoms A:501, A:502 and B:501). While distinguishing sodium ions from water molecules is challenging in many cases due to similar coordination and number of electrons, it is unambiguous in this case. The atoms are six-coordinated with octahedral geometry and bonds in the range 2.3–2.5 Å. This geometry is typical for sodium ions but not for water molecules. CheckMyMetal validation tool^54^ confirms correct ions placement. The overall valencies are close to the unit of valence for these atoms that confirms that sodium ions are more chemically sensible in these positions than water molecules.

### Molecular dynamics simulations and pKa calculations

Chain A of the high salt structure was preprocessed to remove alternative conformations and model missing side chains using the energy-based optimization protocols available in ICM-Pro (v.3.8-7)^55^. Starting pyroglutamic acid and ending His-tag were removed. The preprocessed structures were protonated using GROMACS (v. 2020.1)^56^. Since the true protonation state of His112 at physiological pH is unknown, two different protonation states for His112 at ND1-atom were modeled in two separate 1 µs runs. Molecular dynamics simulation was performed using the OPLS-AA force field with SPC/E model of water. The structure was simulated in a periodic cubic box containing 7972/7983 water molecules and 10/11 chloride ions for uncharged/charged run, respectively. After the initial energy minimization, the system was consequently equilibrated in 100-ps NVT and 100-ps NVT phases with position restraining force on the heavy atoms of the protein, followed by production runs for 1 µs with 2 fs timestep using modified Berendsen thermostat and Parrinello-Rahman barostat for both protonation states. Backbone Root Mean Square Deviation (RMSD) of the after least square fit to the starting model backbone for the production runs are shown on the Supplementary Fig. 2. The simulations were performed using GROMACS (v2020.1) simulation package on a working station with a single Nvidia GTX 1050 GPU. pKa calculations were performed using PROPKA 3.2 within propkatraj package^57,58^ and plotted using seaborn package^59^.

## Supplementary Information

Supplementary Information.

## Data Availability

The structures were deposited to RCSB Protein Data Bank (PDB): https://www.rcsb.org/structure/8BBU (low salt) and https://www.rcsb.org/structure/8BBW (high salt). The respective raw diffraction images were deposited to Integrated Resource for Reproducibility in Macromolecular Crystallography^60^ under corresponding PDB numbers.
