# Supplementary material for: Structural insights into thrombolytic activity of destabilase from medicinal leech
Source: Sci Rep. 2023 Apr 24;13:6641. doi: 10.1038/s41598-023-32459-x (PMC10126035; doi:10.1038/s41598-023-32459-x)
Supplement: Supplementary file 1 — Supplementary Information. [file 41598_2023_32459_MOESM1_ESM.docx]

# Supplementary Information for

# Structural insights into thrombolytic activity of destabilase from medicinal leech

Egor Marin^1,6,&^, Daniil A. Kornilov^1,&^, Sergey S. Bukhdruker^1^, Vladimir A. Aleksenko^1^, Valentin A. Manuvera^1,2^, Egor V. Zinovev ^1^, Kirill V. Kovalev^3^, Mikhail B. Shevtsov^1^, Anna A. Talyzina^1,5^, Pavel A. Bobrovsky^1,2^, Pavel K. Kuzmichev^1^, Alexey V. Mishin^1^, Ivan Y. Gushchin^1^, Vassili N. Lazarev^1,2^, Valentin I. Borshchevskiy^1,4,*^

^1^ Moscow Institute of Physics and Technology, Dolgoprudny, Russia

^2^ Lopukhin Federal Research and Clinical Center of Physical-Chemical Medicine of Federal Medical Biological Agency, Moscow, Russia

^3^ EMBL Outstation Hamburg, c/o DESY, Hamburg, Germany

^4^ Joint Institute for Nuclear Research, Dubna, 141980, Russian Federation

^5^ Present address: Department of Molecular Biosciences, Northwestern University, Evanston, IL, USA

^6^ Present address: Groningen Biomolecular Sciences and Biotechnology Institute, University of Groningen, Groningen, The Netherlands

^&^ – equal contribution

* – corresponding author

| **Supplementary Table 1.** **Crystallographic data collection and refinement statistics.**  Data Collection Statistics | | |
| --- | --- | --- |
| Name | High salt | Low salt |
|  | High Na^+^ concentration | Low Na^+^ concentration |
| PDB ID | 8BBU | 8BBW |
| Source | ESRF ID23-1 | ESRF ID29 |
| Detector | PILATUS 6M-F | PILATUS 6M-F |
| Wavelength (Å) | 0.972 | 1.072 |
| Space group | P1 | P2_1_ |
| Cell dimensions |  |  |
| a, b, c (Å) | 27.4, 36.2, 61.4 | 26.4, 35.9, 54.5 |
| α, β, γ (°) | 106.8, 92.6, 95.5 | 90, 92.3, 90 |
| No. of observations | 299863 (27712) | 129567 (11931) |
| No. of unique reflections | 82400 (7805) | 19400 (1884) |
| Resolution (Å) | 34.40 - 1.10  (1.14 - 1.10) | 27.21 - 1.4  (1.45 - 1.4) |
| Rmeas (%) | 8.0 (69.4) | 12.2 (49.7) |
| Rpim (%) | 4.1 (36.0) | 4.7 (58.5) |
| I/σI | 9.2 (1.7) | 9.5 (1.2) |
| CC1/2 (%) | 99.8 (70.5) | 99.8 (50.2) |
| Completeness (%) | 90.5 (85.7) | 95.8 (93.2) |
| Redundancy | 3.6 (3.6) | 6.7 (6.3) |
| Mosaicity (°) | 0.05 | 0.23 |
| Wilson B-factor (Å) | 9.4 | 13.9 |
| Refinement Statistics | | |
| Resolution (Å) | 34.4 - 1.1  (1.14 - 1.1) | 27.21 - 1.4  (1.45 - 1.4) |
| No. of reflections (work/free) | 82391/1990 (7805/187) | 19397/972  (1884/96) |
| R_work_/R_free_ | 0.125/0.155 | 0.154/0.193 |
| CC_work_/CC_free_ (%) | 97.8/97.5 | 97.2/95.6 |
| No. of atoms |  |  |
| Protein | 2008 | 1016 |
| Sodium | 3 | 0 |
| Solvent | 393 | 132 |
| B-factors (Å^2^) |  |  |
| Protein | 12.0 | 17.3 |
| Sodium | 23.9 | - |
| Solvent | 27.1 | 32.3 |
| No. of TLS groups | - | 9 |
| R.m.s.d |  |  |
| Bond lengths (Å) | 0.008 | 0.014 |
| Bond angles (°) | 1.16 | 1.40 |
| Ramachandran statistics |  |  |
| Favoured (%) | 98.27 | 100 |
| Allowed (%) | 1.73 | 0 |
| MolProbity score | 1.12 | 1.48 |


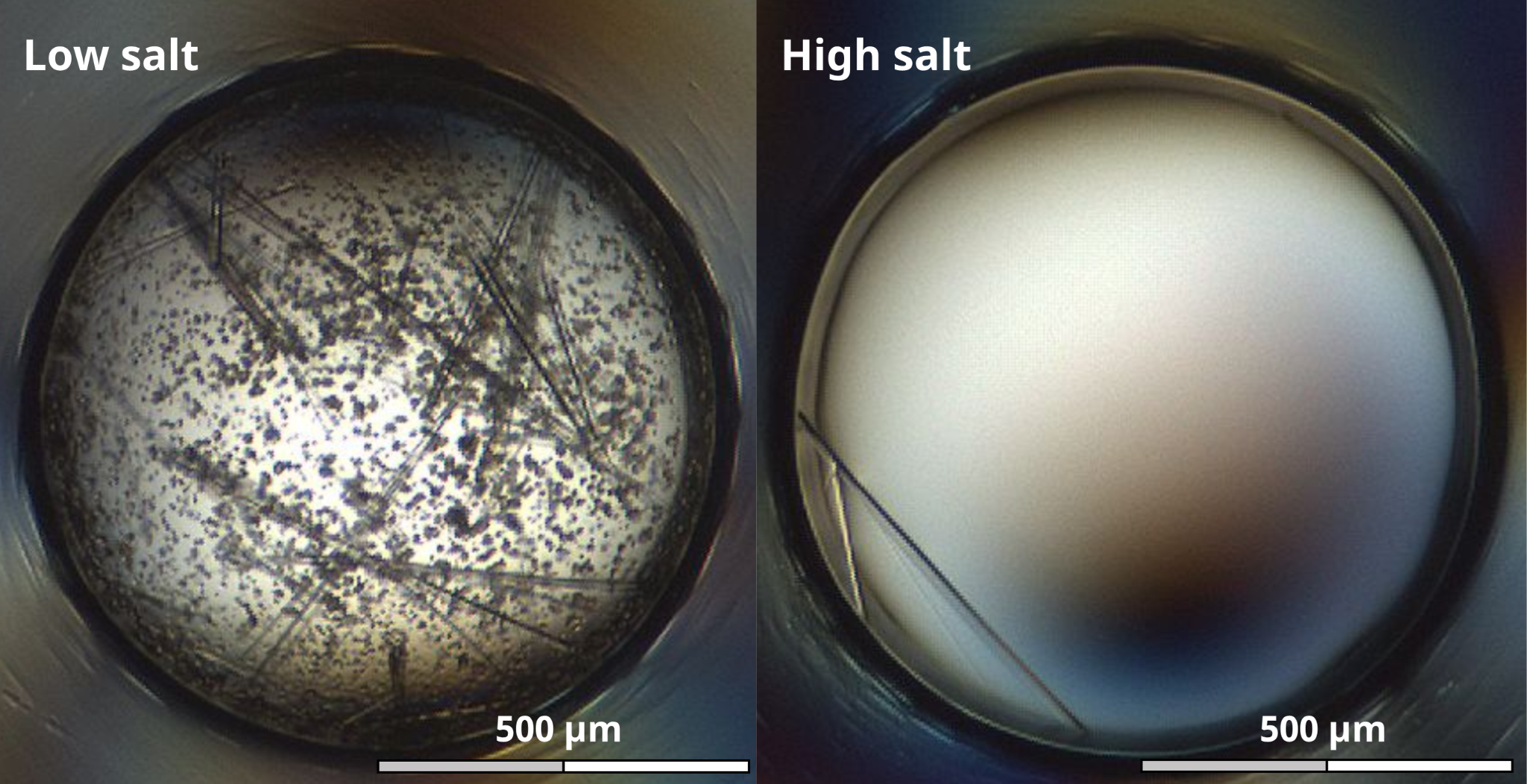


**Supplementary Figure 1.** **Low and high salt destabilase crystals.**

**
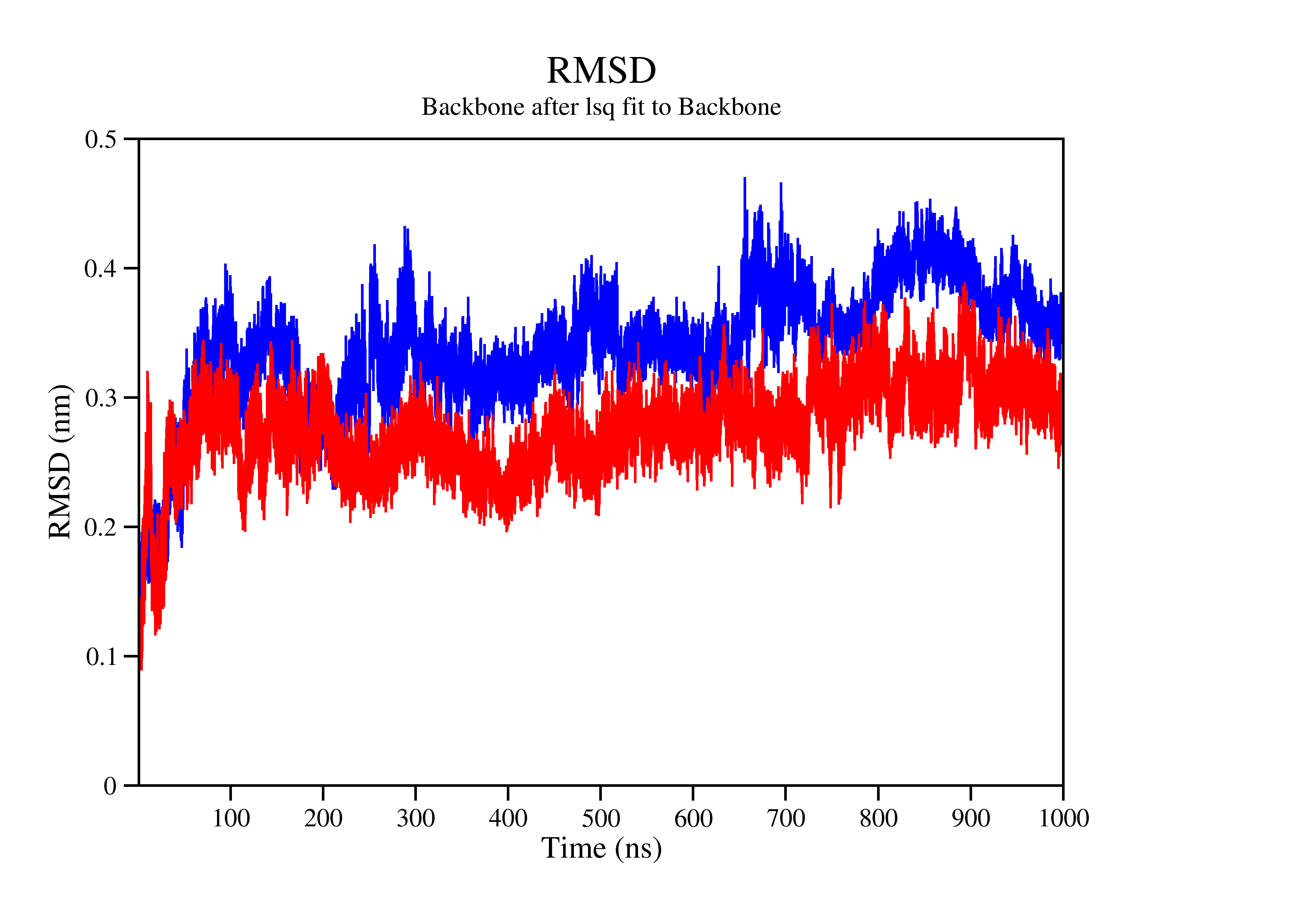
**

**Supplementary Figure 2. Backbone Root Mean Square Deviation (RMSD) of the backbone atoms after least squares fit to the starting model backbone.** Blue and red curves correspond to trajectories with protonated and deprotonated ND1-atom of His112.
